# Embolic Stroke as a Complication of *Streptococcus gordonii* Prosthetic Valve Endocarditis in a Pacemaker Carrier: First Case From Lebanon

**DOI:** 10.1002/ccr3.71389

**Published:** 2025-10-31

**Authors:** Wassim Hamadeh, Yasser A. Harb, Axam Abdelhadi

**Affiliations:** ^1^ Faculty of Medical Sciences Lebanese University Hadath Lebanon; ^2^ Faculty of Medical Sciences Balamand University Balamand Lebanon; ^3^ Mount Lebanon Hospital University Medical Center Hazmieh Lebanon

**Keywords:** embolic stroke, endocarditis, infective endocarditis, prosthetic valve, streptococcal infection, *Streptococcus gordonii*, *Streptococcus gordonii*‐associated endocarditis

## Abstract

In this article, we report the case of a 57‐year‐old hypertensive man with a prosthetic valve and a pacemaker who presented after many hours of onset of left‐sided hemiplegia, left facial drop and dysarthria. Computed tomography of the brain revealed an ischemic stroke, and after an extensive workup the diagnosis of an embolic stroke induced by a *Streptococcus gordonii* endocarditis was established. This organism is considered to be a rare cause of endocarditis as only a dozen cases were previously reported in the literature. We describe the clinical interventions used, expose the diagnostic approach and challenges faced. To the best of our knowledge, this is the first *Streptococcus gordonii*‐associated endocarditis that is reported in Lebanon and, as such, it widens the geographical setting in which it had been reported and helps gain a better understanding of its epidemiology and strain resistance. Furthermore, it highlights the need to include in the differential diagnosis of endocarditis unusual causative and infectious agents, and as such, it urges clinicians to maintain vigilance towards them and take timely intervention against them in order to ameliorate patients' prognosis. We also provide updates in this article on recent research findings and prevention strategies that may be used in the future against infectious endocarditis.

**Summary**

- This case highlights the need to remain highly alert to the possibility of unusual infectious agents in cases of endocarditis in order to start timely and appropriate antibiotic therapy early on, treat aggressively aiming to avoid life‐threatening complications, reduce morbidity and consequently achieve a better prognosis.

## 1 Introduction

Infective Endocarditis (IE) is the result of an established focus of infection in the heart affecting its innermost lining, the endocardium. Despite the significant advances made over the past year in medical treatments and surgical interventions it remains a significant challenge for clinicians as its mortality rate has not changed over 2 decades [1, 2]. Its complications, ranging from abscess formation, valve destruction, embolic events, hemodynamic disorders and heart failure, to name only a few, can be potentially disastrous and involve every organ system [2, 3, 4]. In developed countries, *Staphylococcus aureus* is the leading microorganism incriminated followed by Streptococci and various other pathogens such as enterococci [2]. *Streptococcus gordonii* is a gram‐positive, unencapsulated organism that is a commensal of mucosal surfaces and skin in humans. It has been implicated in a wide variety of opportunistic infections, both in systemic and local diseases, such as septic arthritis, periodontitis, abscesses, peritonitis but rarely reported as a causative agent of endocarditis [5].

Herein, we will expose the case of a man who presented with an embolic stroke. After an extensive workup he was found to have a *Streptococcus gordonnii*‐associated endocarditis. The detailed clinical scenario and therapeutical approach are discussed.

## 2 Case History/Examination

**FIGURE 1:**
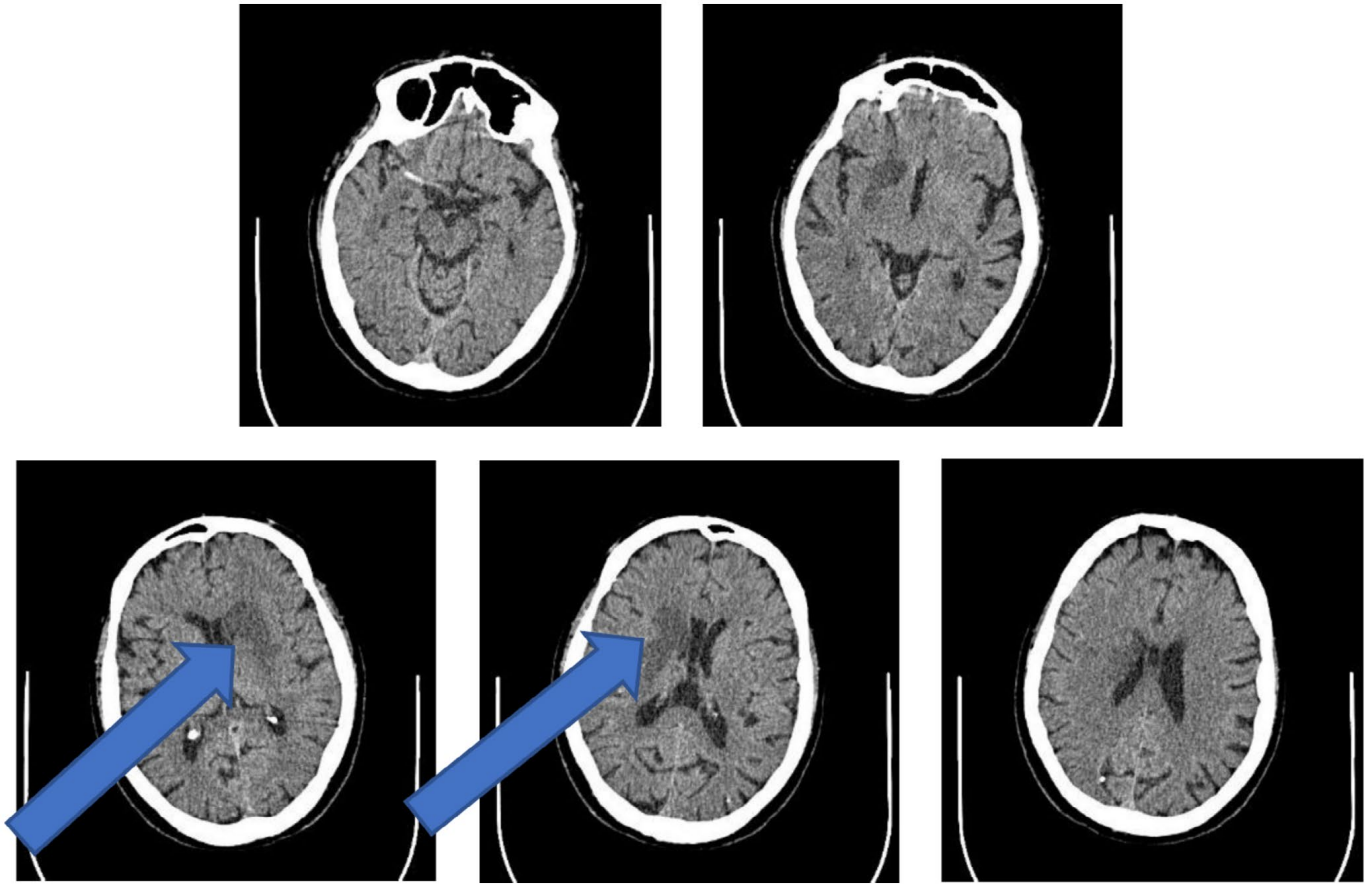
CT scan of the brain showed a 4.6 cm deep parenchymal hypodensity in the region of the right basal ganglia, suggestive of a subacute ischemic infarct (pointed by the arrow). No intracranial bleed, midline shift, hydrocephalus or masses were noted. Those findings in the clinical context were suggestive of an embolic stroke.

A 58‐year‐old ex‐smoker male patient presented to the Emergency Department with left‐sided hemiplegia, a left facial drop, and dysarthria that started abruptly 7 h ago. Upon presentation, he was mildly agitated but not in acute distress and looked well nourished. His vital signs included a blood pressure of 117/76, 85 beats per minute and a low‐grade fever. Upon further questioning, he mentioned that this fever had been continuously present since yesterday. He denied any recent hospitalization, upper respiratory tract infection, recent dental procedure or intravenous drug abuse. His past medical history is most relevant for hypertension (controlled) in the last 12 years for which he has been treated with losartan and dyslipidemia, treated with atorvastatin. Additionally, he was operated on for an aortic valve replacement (AVR) with a biological valve insertion 10 years ago. However, shortly after surgery, he developed a complete atrioventricular block post replacement and, consequently, a dual chamber pacemaker was inserted back then. On physical exam lung fields were clear and a regular rhythm with an aortic systolic click was noted. Neurologically speaking, he was conscious and oriented with a Glasgow coma scale of 15, and a motor power of 4/5 in his affected limbs. Oral exam did not reveal any bleeding, mucositis, periodontal disease or dental caries. A computed tomography (CT scan) of the brain was urgently done: it showed a deep parenchymal hypodensity in the right basal ganglia, a finding compatible with an embolic stroke and the symptomatology (Figure 1). Laboratory results on admission were relevant for leukocytosis, a high C‐reactive protein (CRP) and positive troponin (Table 1). Blood cultures were also taken. Latterly, the patient was admitted to the intensive care unit (ICU) and work up for embolic stroke causes was initiated.

**TABLE 1:** Laboratory tests results on admission demonstrating leukocytosis, neutrophilia, elevated troponin along with a markedly increased C‐reactive protein (CRP) levels.

Hematology		
Hemoglobin	13.5 g/dL	13.0–17 g/dL
Hematocrit	40.9%	40%–45%
**White blood cells**	**13.27 × 10** ^**3**^ **/μL**	4–10 × 10^3^/μL
Neutrophils	81.5%	40%–67%
Lymphocytes	11.7%	20%–40%
INR	1.1	
Chemistry		
Creatinine	0.6 mg/dL	0.60–1.30 mg/dL
**Troponin**	**0.098 ng/mL**	0.00–0.03 ng/mL
Immunology		
**C‐reactive protein (CRP)**	**185 mg/L**	0–5

## 3 Differential Diagnosis

**FIGURE 2:**
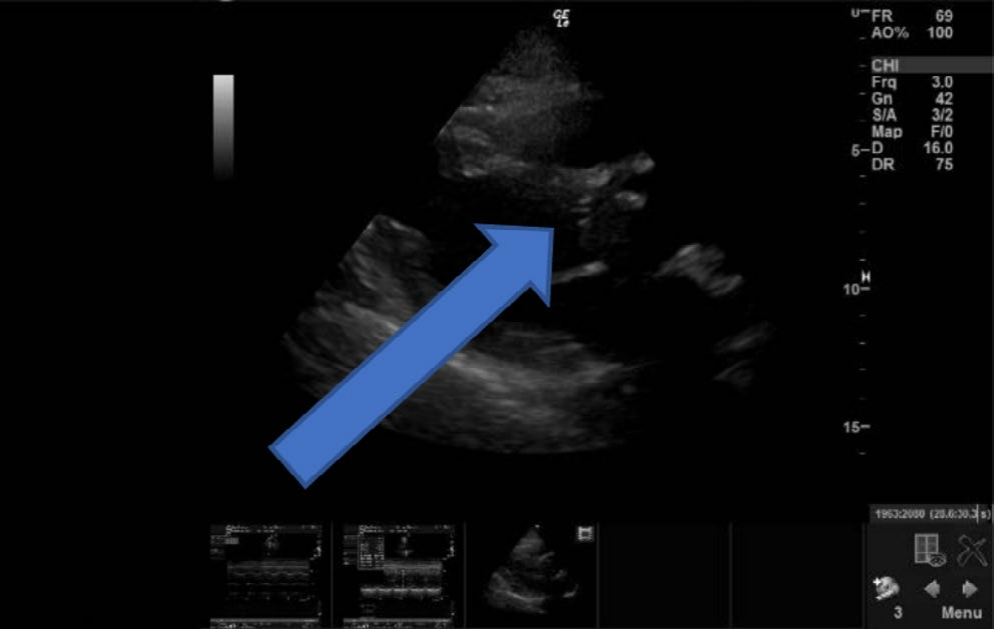
TTE showed aortic valve calcifications with probable vegetations (pointed by the arrow).

At this stage, differential diagnosis included paroxysmal atrial fibrillation, carotid stenosis with plaque embolization, and IE, among others. A Doppler echography of the carotid arteries did not reveal significant stenosis. Furthermore, transthoracic echocardiography (TTE) and telemetry did not show arrhythmias. However, there was an increased suspicion of possible vegetations (Figure 2).

## 4 Conclusion and Results (Outcome and Follow‐Up)

**FIGURE 3:**
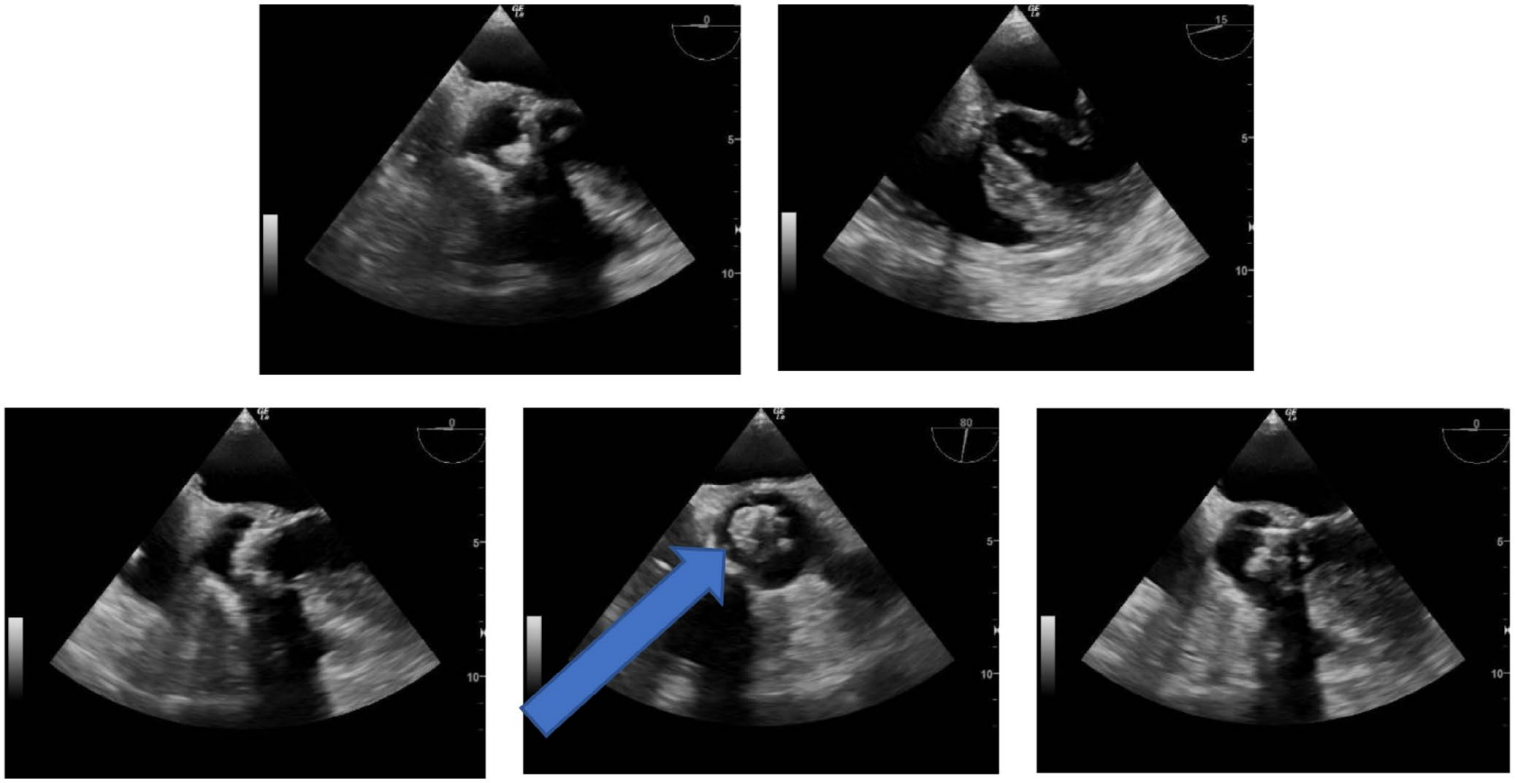
Transesophageal echocardiography showed a thickened biologic aortic valve with probable calcification of the cusps on its tip. The presence of a mobile hyper‐echoic image on a leaflet in favor of vegetations (pointed by the arrow) also is to be noted.

Since TTE raised suspicion of aortic valve vegetations, a transesophageal echocardiography (TEE) was subsequently performed. It confirmed their presence (Figure 3). Giving the clinical scenario, the imaging and laboratory results, and according to the 2023 European Society of Cardiology (ESC) guidelines for the diagnosis and treatment of infective endocarditis and the diagnostic elements of the latest revision of the Duke–ISID criteria, the diagnosis of IE was established. Subsequently, the patient started to be treated with vancomycin and gentamicin. Eventually, after 24 h of incubation, blood cultures revealed the presence of multi‐sensitive *Streptococcus gordonii* and vancomycin was switched to teicoplanin. Blood cultures taken 48 h after antibiotic initiation showed no growth, and inflammatory markers started to decrease progressively. Afterwards, a cardiothoracic surgeon was consulted and the prosthetic valve was removed and replaced with a mechanical one. The patient had repeated transesophageal echocardiography in order to follow up on the vegetation size after 4 weeks from antibiotic initiation. Vegetation size was unchanged. He was discharged home in fair clinical condition after 50 days of hospitalization.

## 5 Discussion

*Streptococcus gordonii* is considered to be a rare causative agent of infective endocarditis: In fact, only 25 cases were found to be documented in the English literature that screened for such cases of *S. gordonii* endocarditis in Embase, PubMed, and Cochrane Library databases [6]. Because different microorganisms have different virulence factors which may lead to different prophylaxis, complications, and treatment regimens, reporting those cases where unusual causative pathogens were incriminated is important: a body of evidence can be formed and lead to a deeper understanding of the associated epidemiology, the patients at risk, the strain's resistance and their geographical distribution. It could definitely in turn lead to a better awareness concerning those unusual microorganisms, consequently lead to risk stratification according to patients' risk factors, clinical history and comorbidities, and, ultimately, treatment recommendation guidelines taking into account the possibility of the presence of those infectious organisms when treating empirically. This is the first case to be reported in Lebanon, and despite the fact that until this date reported cases of *Streptococcus gordonii* endocarditis are rare, it contributes to widening the geographical setting where this etiological agent of endocarditis has previously been reported.

*Streptococcus gordonii* possesses several virulence factors that not only permit its attachment to the endocardial surface of the heart, but also promote inflammation, biofilm and vegetation formation. Understanding those virulence‐mediated pathways could definitely lead to developing better therapeutic arsenals against them. First, like many other microorganisms, the production of biofilms makes it particularly challenging to control the infectious source in vivo by antibiotics [7]. Biofilms should not be thought of as static structures solely made up of pathogens embedded in a self‐produced extracellular matrix against hostile chemical or physical environments and antibiotics but rather as dynamic structures facilitating the exchange of signaling molecules, nutrients and genetic materials between the bacterial community they contain [8]. The challenges are to reach the microorganisms embedded in this self‐produced matrix and to maintain adequate drug concentrations able to exert a therapeutic effect despite the periodic washing by saliva. Up to this day, conventional antimicrobial therapy has been a cornerstone in the management of IE; however, novel therapeutic arsenals are emerging: Novel antibiofilm drugs such as nano drug delivery systems, Polymer‐Based Antimicrobial Delivery Systems, and liposomes are currently under investigation to overcome the challenges associated with biofilms [7]. Furthermore, a potential vaccine against the surface‐located *S. gordonii* proteins Hsa and PadA which are implicated in platelet adhesion and aggregation, a fundamental step in the IE pathogenesis, has shown promising results in order to prevent IE by mounting an early robust immune attack against them [9].

As previously mentioned, this organism is a commensal of the mucosal surfaces. Consequently, bacteremia is thought to arise from a breach or weakening of the oral mucosa for bacterial entry by for instance aggressive dental procedures, oral bleeding or mucositis. However, this was not the case for our patient. The sustained bacterial translocation gives rise to bacteremia and the ensuing cycles of bacterial proliferation on damaged or prosthetic valves or congenital heart abnormalities which constitute a fertile soil for the establishment of an infective focus in the heart, leading to inflammation, monocyte recruitment and thrombosis, ultimately leading to the formation of a mature vegetation. Moreover, the cell wall components contain lipoteichoic acids, lipoproteins, peptidoglycans, beside other cell wall components and when released are believed to overwhelm the patient's immune response and induce further damage [6].

The diagnosis of IE relies mainly on Duke's criteria which integrate risk factors, imaging and laboratory findings; but, unfortunately, it is impossible to differentiate between *S. gordonii‐*associated endocarditis and an IE caused by other organisms based solely on these criteria and the clinical symptomatology [10, 11]. Consequently, when IE is among the differential diagnoses, no antibiotic therapy should be initiated without prior cultures. Even though negative cultures do not exclude the possibility of IE because some pathogens require fastidious medium to grow, or intracellular organisms might be the incriminated agents. Moreover, the symptoms and signs of IE greatly lack specificity such as fever, weight loss, anorexia and fatigue, to mention a few. As a result of being alert to the possibility of IE based on the clinical scenario, the physical exam and history taking, the patient's age, risk factors age and comorbidities, it is vital to start early on and reduce complications. Previous studies have emphasized this role of timely intervention in reducing thromboembolic complications. This true diagnostic complexity of IE associated with the life‐threatening and severe morbidity it carries, should push the clinician to remain highly alert and include it in the differential diagnosis whenever needed. Artificial intelligence might in the future serve as a tool in order to help clinicians personalize the diagnostic approach and tailor treatment, to distinguish patients who might benefit from different types of treatment in different clinical settings.

## 6 Conclusion

This case highlights the need to remain highly alert to the possibility of unusual infectious agents in the case of endocarditis in order to start with timely and appropriate antibiotherapy early on, treat aggressively aiming to avoid life‐threatening complications, reduce morbidity and consequently achieve a better prognosis. A multidisciplinary approach involving cardiologists, cardiothoracic surgeons, infectious diseases specialists, neurologists and radiologists is often required to promptly manage infective endocarditis and its complications. Further research is needed to unravel the virulence‐mediated pathways of organisms, the factors that enable them to establish an infective focus in the heart and develop effective anti‐biofilm strategies, in order to treat effectively and even prevent those infections and their life‐threatening complications.

## Author Contributions

**Wassim Hamadeh:** conceptualization, investigation, supervision, validation, visualization, writing – original draft, writing – review and editing. **Yasser A. Harb:** conceptualization, investigation, validation, visualization, writing – original draft. **Axam Abdelhadi:** investigation, supervision, validation, visualization.

## Ethics Statement

Ethical approval was obtained from Mount Lebanon Hospital for this case to be published on May 26, 2025.

## Consent

The patient provided a written informed consent for his case to be published and is available for the journal editor upon request.

## Conflicts of Interest

The authors declare no conflicts of interest.

## Data Availability

The authors have nothing to report.

## References

[1] S. Modan , K. Madan , G. Mugwagwa , et al., “An Epidemiological Update on Infective Endocarditis: A Two‐Decade Retrospective Longitudinal Analysis,” European Heart Journal 45, no. Supplement 1 (2024): ehae666.2620, 10.1093/eurheartj/ehae666.2620.

[2] T. J. Cahill , L. M. Baddour , G. Habib , et al., “Challenges in Infective Endocarditis,” Journal of the American College of Cardiology 69, no. 3 (2017): 325–344, 10.1016/j.jacc.2016.10.066. 28104075

[3] C. Selton‐Suty , F. Goehringer , C. Venner , et al., “Complications et Pronostic de l'Endocardite Infectieuse [Complications and Prognosis of Infective Endocarditis],” Presse Médicale 48, no. 5 (2019): 532–538, 10.1016/j.lpm.2019.04.002. 31056233

[4] J. M. Ferro and A. C. Fonseca , “Infective Endocarditis,” Handbook of Clinical Neurology 119 (2014): 75–91, 10.1016/B978-0-7020-4086-3.00007-2. 24365290

[5] O. J. Park , Y. Kwon , C. Park , et al., “ *Streptococcus gordonii*: Pathogenesis and Host Response to Its Cell Wall Components,” Microorganisms 8, no. 12 (2020): 1852, 10.3390/microorganisms8121852. 33255499 PMC7761167

[6] G. A. Ali , A. Pérez‐López , C. Tsui , et al., “ *Streptococcus gordonii* ‐Associated Infective Endocarditis: Case Series, Literature Review, and Genetic Study,” Clinical Case Reports 12 (2024): e8684, 10.1002/ccr3.8684. 38585580 PMC10996068

[7] I. Kanwar , A. K. Sah , and P. K. Suresh , “Biofilm‐Mediated Antibiotic‐Resistant Oral Bacterial Infections: Mechanism and Combat Strategies,” Current Pharmaceutical Design 23 (2017): 2084–2095. 27890003 10.2174/1381612822666161124154549

[8] W. Yin , Y. Wang , L. Liu , and J. He , “Biofilms: The Microbial ‘Protective Clothing’ in Extreme Environments,” International Journal of Molecular Sciences 20, no. 14 (2019): 3423, 10.3390/ijms20143423. 31336824 PMC6679078

[9] S. Mancini , C. Menzi , F. Oechslin , P. Moreillon , and J. M. Entenza , “Antibodies Targeting Hsa and PadA Prevent Platelet Aggregation and Protect Rats Against Experimental Endocarditis Induced by *Streptococcus gordonii* ,” Infection and Immunity 84, no. 12 (2016): 3557–3563, 10.1128/IAI.00810-16. 27736784 PMC5116732

[10] Z. Dadon , A. Cohen , Y. M. Szterenlicht , et al., “Spondylodiskitis and Endocarditis due to *Streptococcus gordonii* ,” Annals of Clinical Microbiology and Antimicrobials 16 (2017): 68, 10.1186/s12941-017-0243-8. 28978355 PMC5628438

[11] A. Pérez‐Vázquez , M. C. Fariñas , J. D. García‐Palomo , J. M. Bernal , J. M. Revuelta , and J. González‐Macías , “Evaluation of the Duke Criteria in 93 Episodes of Prosthetic Valve Endocarditis: Could Sensitivity Be Improved?,” Archives of Internal Medicine 160, no. 8 (2000): 1185–1191, 10.1001/archinte.160.8.1185. 10789613

